# Registration Difficulties.—IV

**Published:** 1908-11-28

**Authors:** 


### NURSING ADMINISTRATION.

### REGISTRATION DIFFICULTIES.—IV.

DEALING exclusively with nurses trained in voluntary hospitals, it is estimated that a yearly average of about 1,100 nurses annually complete their training in general hospitals containing 100 and more beds in the United Kingdom. It is interesting to examine into the number of nurses under training in special hospitals and in institutions containing fewer than 100 beds. There is first a group of general hospitals containing under 100, but over 40, beds. These institutions are recognised as training schools by the Royal British Nurses' Association, and in any proposed scheme for registration a strong claim would be urged on their behalf for recognition. It is indisputable that the line which severs them from the class above and the class below in numerical strength is very fine. Many hospitals in this class have a high standing, considered as training schools, for the variety and excellence of the work carried on in the wards. Many of them, again, are inferior in every particular to some hospitals, such as the Miller General Hospital whose beds fall far below in number even the estimate of the Royal British Nurses' Association, but which are able to afford good training nevertheless. The following table shows the number of probationers under training in the United Kingdom in various classes of hospitals excluded, under the 100 beds condition, from recognition:—

| | London | Provincial | Scotch | Irish | Total |
|---|---|---|---|---|---|
| Under 100, but over 40 beds | 82 | 556 | 28 | 36 | 702 |
| Under 40 beds | 26 | 250 | 35 | 10 | 321 |
| Special hospitals | 393 | 301 | 79 | 50 | 823 |
| | | | Total | | 1,846 |

Among the special hospitals have been reckoned only those which undertake to give a two or three years' course of training for probationers—namely, children's, women's, consumptive, etc. Maternity hospitals giving only a few months' training have been omitted, and all fever institutions which, since they are of a municipal character, cannot be reckoned among voluntary institutions. It will be seen that omitting all these numerous institutions there are at the present moment under training in the United Kingdom nearly 2,000 nurses whose claim to be admitted to the qualifying examination for registration would be at stake. The number of institutions affected by the exclusion of their probationers from the status of "trained nurse" is very instructive.

Number of institutions training one probationer or more:—

| | London | Provincial | Scotch | Irish | Total |
|---|---|---|---|---|---|
| Under 100, but over 40 beds | 5 | 72 | 6 | 7 | 90 |
| Under 40 beds | 5 | 94 | 15 | 5 | 119 |
| Special hospitals | 31 | 39 | 6 | 8 | 84 |
| | | | Total | | 293 |

It is possible that the regulations for registration framed by the General Nurses' Council would admit the claim of some few of the larger hospitals included in these tables to continue training probationers. The remainder would be reduced to a curiously anomalous position. By no stretch of probability would they be able to subsist on the labour of women prepared to enter upon a three years' course at a larger hospital on the conclusion of their first novitiate. They would still continue to train women, but the causes which at present make in the direction of efficiency would be removed. Do what they might, their nurses would be branded as "untrained," and it cannot be doubted that among hundreds of hospitals there would be set stirring a spirit of resistance to the principle of registration. Many institutions would be driven back to regard merely their own interests, and get their labour as cheap as possible; they would relax the effort to provide lectures and advantages for their nurses, and become entirely indifferent as to their future. On the other hand, hospitals which trained conscientiously, and turned out capable nurses, would naturally depreciate the "registered nurse" and run their own candidates for those who would "never think of inquiring whether a nurse were registered or not." Thus the system which aims at regulating nursing would produce confusion worse confounded. It is well known that the leading advocates of registration are most anxious to fix the standard at "three years' consecutive training in one hospital" of the required number of beds. This is so well understood that it may be regarded as one of the first principles of registration, and since lukewarm people are hardly likely to find places on the Board, this standard is what we are compelled to contemplate as awaiting the nursing world in the event of registration. It is hardly too much to say that it cuts at the root of all special and fever hospitals, and, unless it became a dead letter, would render skilled nursing in such institutions a difficult and delicate problem. The matter lies in a nutshell. Women who have taken two years' special training, whether in fever or other hospitals, are seldom able to take a further three years' course before beginning to earn their living. Even when circumstances admit of their doing so, they shrink from beginning all over again in the position of raw probationers. Nor is it practicable, even were they willing, for in big training schools the matron prefers to mould her probationers from the beginning, and finds the presence of partly trained nurses in the wards an embarrassment. Well, then, it will be said, let the trained nurse take a course of special training at the completion of her three years' course. But no special hospital could possibly be carried on by nurses who came in as pupils merely to gain extra experience. The number of such pupils must always be limited to a few at a time, and the original dilemma remains unsolved. Are the probationers trained in the smaller and special hospitals to be altogether ignored? It is very certain that these hospitals cannot be carried on without probationers, and it must fill with misgivings all who have the interests of nursing at heart to consider that hundreds of institutions may be compelled to go on training women to rank as competitors to the "registered nurse," or possibly be branded in the eyes of the public as "impostors."

